# Vergleich der Anwendung verschiedener extraglottischer Atemwegshilfen durch Laien am Phantom

**DOI:** 10.1007/s00063-021-00834-z

**Published:** 2021-06-14

**Authors:** Christoph Jänig, Oliver Balogh, Stephanie Krappitz, Arnulf Willms, Willi Schmidbauer, Tim Piepho

**Affiliations:** 1 Klinik für Anästhesiologie und Intensivmedizin, Bundeswehrzentralkrankenhaus Koblenz, Rübenacher Str. 170, 56072 Koblenz, Deutschland; 2 Klinik für Allgemein-Viszeral- und Thoraxchirurgie, Bundeswehrzentralkrankenhaus Koblenz, Koblenz, Deutschland; 3 Abteilung für Anästhesie, Krankenhaus der Barmherzigen Brüder, Trier, Deutschland

**Keywords:** Atemwegsmanagement, Laienreanimiation, Larynxmasken, Notfallmedizin, Intubation, Airway management, Basic life support, Laryngeal masks, Emergency medicine, Intubation

## Abstract

**Hintergrund:**

Supraglottische Atemwege (SGA) sind eine etablierte Methode der Atemwegssicherung sowohl in der prähospitalen Medizin als auch im klinischen Umfeld. Die endotracheale Intubation ist der Goldstandard, jedoch bieten SGA Vorteile in Bezug auf die schnellere Erlernbarkeit der Anwendung.

**Ziele:**

In der vorliegenden Studie wurde untersucht, ob sich die Zeit bis zur ersten suffizienten Beatmung bei 3 untersuchten supraglottischen Atemwegen, angewendet durch medizinische Laien an einem Phantom, signifikant unterscheidet.

**Material und Methoden:**

Besucher eines Einkaufszentrums (*n* = 160) wurden nach permutierender Blockrandomisierung einer der 3 SGA zugeordnet. Primärer Endpunkt der vorliegenden Studie war die benötigte Platzierungszeit bis zur ersten suffizienten Beatmung.

**Ergebnisse:**

Die Platzierung der i‑gel-Larynxmaske (Intersurgical Beatmungsprodukte GmbH, Sankt Augustin, Deutschland) gelang den Teilnehmern im Median nach 11 s, wohingegen die Zeitspanne bis zur ersten suffizienten Beatmung mittels klassischer Larynxmaske (LMA; 26 s), respektive Larynxtubus (LT; 28 s) im Median deutlich länger war. Damit war die Zeitersparnis bei der Anwendung der i‑gel im Vergleich zu LT und LMA jeweils signifikant größer (*p* < 0,001), wohingegen sich die Zeiten zwischen LT und LMA nicht signifikant unterschieden (*p* 0,65).

**Schlussfolgerung:**

Die Ergebnisse zeigen, dass Laien in der Lage sind, verschiedene supraglottische Atemwege nach einer kurzen Lernphase erfolgreich am Phantom anzuwenden. Die i‑gel-Larynxmaske konnte in unserem Setting mit der höchsten Erfolgsrate und höchsten Geschwindigkeit platziert werden.

## Abstract (en)

**Background:**

Supraglottic airways (SGA) are an established method of airway management both in prehospital medicine and clinical settings. Endotracheal intubation is the gold standard, but SGA offer advantages in terms of faster application learnability.

**Objectives:**

In the present study it was investigated whether the time until the first sufficient ventilation in the three examined SGAs applied by bystander differed significantly.

**Materials and methods:**

A total of 160 visitors to a shopping mall were assigned to one of the three SGA after permutative block randomization. The primary endpoint of the present study was the required placement time until the first sufficient ventilation.

**Results:**

Participants managed to place the i‑gel laryngeal mask airway (i-gel, Intersurgical Beatmungsprodukte GmbH, Sankt Augustin, Germany) after a median time of 11 s, whereas the median time until the first sufficient ventilation using a classic laryngeal mask airway (LMA; 26 s) or a laryngeal tube (LT; 28 s) was significantly longer. Thus, the time savings when using the i‑gel compared to the LT and LMA were each significant (*p* < 0.001), whereas the times between LT and LMA did not differ significantly (*p* 0.65).

**Conclusion:**

The results show that laypersons are able to successfully apply various supraglottic airways to the phantom after a short learning period. The i‑gel laryngeal mask could be placed with the highest success rate and speed.

## Einleitung

Die suffiziente Oxygenierung des Patienten ist eine fundamentale Maßnahme im Herz-Kreislauf-Stillstand und trägt in der Notfallmedizin essenziell dazu bei, die Überlebensrate sowie das neurologische Outcome der Patienten zu verbessern [19, 23]. Als Goldstandard hat sich die endotracheale Intubation (ETI) etabliert [7, 8]. Diese sollte jedoch nur von erfahrenen Anwendern durchgeführt werden, da Fehlintubationen mit fehlender Erfahrung zunehmen und selbst nach Erkennen und Korrektur dieser die ETI dann mit einer deutlich erhöhten Mortalität einhergeht [5].

Für weniger geschultes Personal und für Laien haben sich in vergangenen Studien dagegen supraglottische Atemwege als mögliche Alternative erwiesen, da diese einfacher zu platzieren sind und dennoch einen, wenn auch eingeschränkten Aspirationsschutz bieten [6].

Da bis zum Eintreffen des Rettungsdienstes oftmals Zeiten von 8–15 min überbrückt werden müssen, hängt ein gutes neurologisches Outcome und nicht zuletzt das Überleben der Patienten in Reanimationssituationen von der suffizienten Laienreanimation ab. Dabei zeigen sich jedoch gerade in Bezug auf die Beatmung des Patienten regelmäßig Ressentiments (wie z. B. Panik, die Angst, etwas falsch zu machen, oder auch die Angst vor Infektionen oder Ekel vor Schleim und Erbrochenem; [21]). Resultierend daraus wird in 72 % der Herz-Kreislauf-Stillstände nicht durch Laien reanimiert [1]. Durch den Einsatz von supraglottischen Atemwegshilfen könnte hier Berührungsängsten und hygienischen Bedenken begegnet werden.

Die durchgeführte Studie untersucht, ob es eine supraglottische Atemwegshilfe (SGA) gibt, die im Vergleich zu anderen SGA durch in der Atemwegssicherung unerfahrene Anwender schneller und sicherer am Phantom platziert werden kann.

## Material und Methoden

Im Rahmen der „Woche der Wiederbelebung“ 2018 wurden Besucher eines Einkaufszentrums in Maßnahmen des Basic Life Support (BLS) und der AED(Automatischer Externer Defibrillator))-Anwendung geschult. Anschließend erfolgte die Rekrutierung für das Studienvorhaben. Ein Alter unter 18 Jahren wurde als Ausschlusskriterium für die Aufnahme in die Studie angesehen. Andere Ausschlusskriterien existierten nicht, sodass alle Passanten nach schriftlicher Einwilligung partizipieren konnten. Insgesamt wurden 160 Probanden in die Studie eingeschlossen. Mittels permutierender Blockrandomisierung wurden die Teilnehmer einer von 3 Versuchsgruppen entsprechend der zu verwendenden SGA zugeordnet. Zum Vergleich standen der Larynxtubus-Suction Disposable (VBM Medizintechnik, Sulz a.N., Deutschland), Larynxmaske AuraOnce© (Ambu GmbH, Bad Nauheim, Deutschland) und die Larynxmaske i‑gel© (Intersurgical Beatmungsprodukte GmbH, Sankt Augustin, Deutschland).

**Figure 1:**
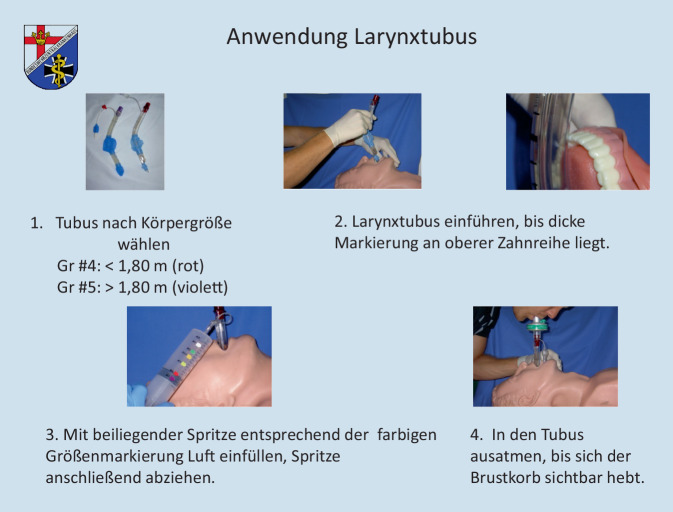

Nach einer kurzen standardisierten, praktischen Anwendungsdemonstration am Phantom durch stets den gleichen Mitarbeiter des Studienteams, bei der explizit die Punkte Platzierung und Einführtiefe anhand einer bebilderten Anleitung (Abb. 1) erläutert wurden, erfolgte der Platzierungsversuch unter Aufsicht eines Mitglieds des Studienteams an einem Modell (AmbuMan Advanced®, Ambu GmbH, Bad Nauheim, Deutschland).

Primäre Zielvariable war die Platzierungszeit der einzelnen SGA. Sekundäre Zielvariablen stellten die Ersterfolgsrate, die Anzahl notwendiger Lagekorrekturen, die Häufigkeiten auftretender Anwendungsprobleme sowie der Einfluss einer medizinischen Berufsausbildung auf den Platzierungserfolg dar.

Die Platzierungszeit wurde ab dem Zeitpunkt des Aufnehmens der SGA bis zur ersten suffizienten Beatmung mittels Beatmungsbeutel (Ambu SPUR II®, Ambu GmbH, Bad Nauheim, Deutschland) gemessen. Ein Platzierungsversuch galt als erfolgreich, wenn die Anwendung der SGA mit einer suffizienten Beatmung endete. Es durften maximal 2 Lagekorrekturen durchgeführt werden. Eine Zeitvorgabe wurden den Teilnehmern nicht gemacht.

Im Anschluss an die praktische Versuchsdurchführung ging ein Mitglied des Studienteams mit den Teilnehmern einen standardisierten Fragebogen durch, um Aspekte der Handhabung und eine subjektive Bewertung zur Beatmung mittels SGA abzugeben.

Die Daten wurden mittels Excel (Microsoft Corporation, Redmond, Washington State, USA) erfasst und mit der Statistiksoftware IBM SPSS Statistics 26.0 (IBM Corp., Armonk, NY, USA) in Zusammenarbeit mit dem Institut für medizinische Biometrie, Epidemiologie und Informatik, Abteilung für Biometrie und Bioinformatik der Johannes-Gutenberg-Universität Mainz ausgewertet.

Die Anzahl der Probanden wurde für eine statistische Power von 80 % (entsprechend β‑Fehler 0,2) und einen α‑Fehler von 0,05 adaptiert. Als statistisch signifikant wurden *p*-Werte ≤ 0,05 betrachtet. Dabei war die Nullhypothese, dass zwischen den einzelnen SGA kein Unterschied in Bezug auf die Platzierungszeit besteht. Kategoriale Variablen wurden anhand von prozentualen Anteilen und Häufigkeiten charakterisiert. Ihre statistische Signifikanz wurde durch Anwenden des χ^2^-Tests, respektive des exakten Fisher-Tests bestimmt, da es sich bei den Daten ausschließlich um voneinander unabhängige Stichproben handelt. Stetige Variablen wurden bei Vorliegen einer Normalverteilung durch Mittelwerte und Standardabweichung beschrieben; lag keine Normalverteilung vor, kamen deskriptiv der Median und Interquartilsabstände zum Einsatz. Da es sich auch bei den stetigen Variablen gleichzeitig um unverbundene Werte handelt, wurde zum Vergleich der normalverteilten Werte der unverbundene t‑Test nach Welch, bzw. beim Vergleich von mehr als 2 Gruppen der ANOVA-Test benutzt. Da die Platzierungszeiten der einzelnen SGA nicht normalverteilt waren, wurden entsprechend nichtparametrische Tests, namentlich der Mann-Whitney-U-Test beim Vergleich von 2 Gruppen sowie der Kruskal-Wallis Test bei mehr als zwei verglichenen Gruppen, verwendet.

## Ergebnisse

An der vorliegenden Untersuchung nahmen insgesamt 160 Probanden teil, von denen 75 (46,9 %) männlich und 83 (51,9 %) weiblich waren. Zwei Probanden machten keine Angaben über ihr Geschlecht (1,3 %). Insgesamt machten 146 Probanden Angaben zu ihrem beruflichen Hintergrund. Von diesen besaßen 74,7 % (*n* = 109) keinerlei medizinische Vorbildung. 25,3 % (*n* = 37) übten dagegen einen Beruf im Gesundheitssystem (Kranken- und Altenpflege, Rettungsassistent und -sanitäter) aus. 53 Probanden wurden der i‑gel-Gruppe, 51 der LMA(Larynxmaske)-Gruppe und 56 der LT(Larynxtubus)-Gruppe zugeordnet.

Die Platzierung der i‑gel gelang den Teilnehmern im Median nach 11 s, wohingegen die Zeitspanne bis zur ersten suffizienten Beatmung mittels LMA (26 s), respektive LT (28 s) im Median deutlich länger war. lag. Damit war die Zeitersparnis bei der Anwendung der i‑gel im Vergleich zu LT und LMA jeweils signifikant schneller (*p* < 0,001), wohingegen sich die Zeiten zwischen LT und LMA nicht signifikant unterschieden (*p* 0,687; Tab. 2).

**Figure 2:**
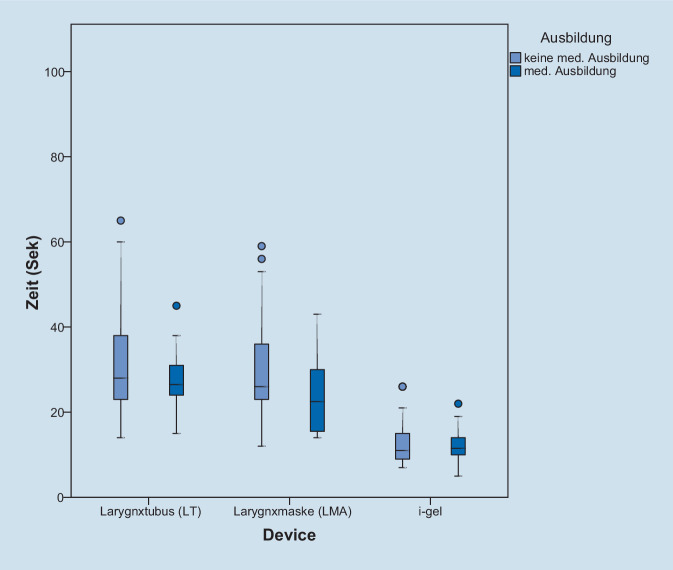

Aufgrund der geringen Probandenzahl in der Subgruppe mit medizinischer Vorbildung konnte kein statistisch auffälliger Unterschied in den Platzierungszeiten zwischen medizinischem Personal und nicht medizinisch ausgebildeten Helfern erzielt werden (Abb. 2, Tab. 1).

**Table 1:** 

	Zeit bis zur erfolgreichen Platzierung		Signifikanz
	Median (±SA [s])		
	Med. Ausbildung	Keine Med. Ausbildung	Med. Ausbildung/
			Keine med. Ausbildung
LT	27,5 (10,41)	27 (15,35)	*p* 0,869
LMA	22,5 (10,18)	26 (15,16)	*p* 0,153
i‑gel	11,5 (4,62)	11 (7,28)	*p* 0,1

*LT* Larynxtubus, *LMA* Larynxmaske, *i‑gel* i‑gel Larynxmaske, *SA* Standardabweichung

**Table 2:** 

	Platzierungszeit (Median ± SA [s])		Differenz der Platzierungszeit (s)	Signifikanz
LMA vs. i‑gel	26 (15,5)	11 (6,5)	15	*p* < 0,0001
LT vs. i‑gel	28 (19,8)	11 (6,5)	17	*p* < 0,0001
LT vs. LMA	28 (19,8)	26 (15,5)	2	*p* 0,687

Darüber hinaus zeigte sich kein statistisch auffälliger Unterschied in Bezug auf die Anzahl der benötigten Versuche bis zur ersten suffizienten Beatmung (*p* 0,07) bzw. in Bezug auf die Anzahl der benötigten Lagekorrekturen (*p* 0,58). Die Platzierung im Erstversuch war bei der i‑gel in 80 %, der LMA in 68 % und dem LT in 67 % erfolgreich (*p* 0,07).

**Figure 3:**
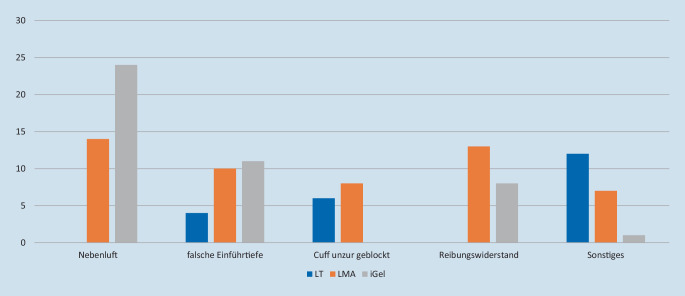

41,5 % der Probanden in der i‑gel-Gruppe gaben Anwendungsprobleme an. Diese traten bei den beiden anderen Gruppen in 23,5 % (LMA) und 37,5 % (LT) der Versuche auf. Die Häufigkeit von Anwendungsproblemen zeigte sich als statistisch ebenfalls nicht auffällig. Es gibt jedoch deutliche Unterschiede zwischen den SGA in Bezug auf die Nebenluft am Phantom. Beim Larynxtubus zeigte sich hier keine Problematik (*n* = 1) bei der Larynxmaske in 27,5 % (*n* = 14), bei der i‑gel in 45,3 %, entsprechend bei 24 Teilnehmern (*p* < 0,001; Abb. 3).

Die subjektive Einschätzung der Probanden bezüglich der Anwenderfreundlichkeit unterschied sich zwischen den einzelnen EGA nicht. 96 % der LT-Anwender (*n* = 51), 100 % der LMA-Anwender (*n* = 50) und 100 % der i‑gel-Anwender (*n* = 48) schätzten deren Anwendung als „leicht“ oder „eher leicht“ ein.

So befürworteten 100 % der Probanden in den Gruppen „LT“ und „i-gel“, dass diese Atemwegshilfen im Rahmen der Erste-Hilfe-Ausbildung ausgebildet werden sollten. Lediglich 4 Anwender der LMA (7,8 %) sprachen sich gegen die Ausbildung im Erste-Hilfe-Kurs aus.

## Diskussion

Die endotracheale Intubation ist weiterhin die Atemwegssicherungsmethode der Wahl für den geübten Anwender. Supraglottische Atemwegshilfen haben seit ihrer Einführung in den klinischen und präklinischen Alltag stark an Bedeutung gewonnen [3, 14, 22].

In den vergangenen Jahren wurden wiederholt Studien durchgeführt, die die Anwendung einer SGA durch Laien am Phantom untersuchten [17, 18]. Es konnte gezeigt werden, dass hohe Platzierungsraten für die einzelnen SGA auch durch Laien erzielt werden konnten.

Vereinbar mit den Ergebnissen der vorliegenden Studie gelang es auch Schälte et al. zu demonstrieren, dass die Anwendung von supraglottischen Atemwegen, insbesondere der i‑gel-Larynxmaske, leicht zu erlernen ist [18]. Dabei stellte sich heraus, dass mithilfe einer modifizierten i‑gel (mit adaptiertem Mundstück) knapp 80 % der Versuchspersonen nach bereits kurzer, ausschließlich schriftlicher Einweisung suffizient ein Phantom beatmen konnten.

Die durchgeführte Studie untersucht im Gegensatz zur Studie von Schälte et al. aus dem Jahr 2011 [17] erstmals 3 nichtmodifizierte, zwischenzeitlich weit verbreitete SGA hinsichtlich der Atemwegssicherung durch unerfahrene Anwender am Phantom unter Einbeziehung der i‑gel-Larynxmaske, die konstruktionsbedingt ohne den fehlerbehafteten Schritt des „Cuff blockens“ verwendet werden kann.

Die primäre Zielvariable der vorliegenden Studie ist die Zeit bis zur ersten suffizienten Beatmung. Es kann gezeigt werden, dass die Platzierungszeit der i‑gel signifikant geringer ist als die des LT und die der LMA. Dieses Ergebnis deckt sich zu großen Teilen mit den Erkenntnissen von de Montblanc et al., die 2014 in einer Metaanalyse die i‑gel mit herkömmlichen Larynxmasken der ersten und zweiten Generation unter verschiedenen Gesichtspunkten bei der Anwendung am anästhesierten Erwachsenen miteinander verglichen [4]. So zeigte sich eine im Durchschnitt signifikant geringere Einführungszeit für die i‑gel im Vergleich zu Larynxmasken der ersten, nicht jedoch im Vergleich zu den Larynxmasken der zweiten Generation. Weiterhin konnte gezeigt werden, dass sich die unterschiedlichen Atemwegshilfen nicht signifikant in Bezug auf die Erfolgsrate beim ersten Einführungsversuch unterschieden; eine Erkenntnis, die sich mit den Ergebnissen der vorliegenden Studie deckt.

In den bisher publizierten Studien wurden die angegebenen Zeiträume uneinheitlich definiert, weshalb eine direkte Vergleichbarkeit nur eingeschränkt besteht. Die in dieser Studie gewählte Zeitspanne wurde extra bewusst vom Zeitpunkt des Aufnehmens des SGA bis zur erfolgreichen Ventilation gewählt, um möglichst genau die Dauer der gesamten Prozedur der Atemwegssicherung abzubilden. Hierdurch soll gewährleistet werden, dass die Prozedurdauer nicht durch einen Definitionsfehler bei der Übertragung auf den Patienten zusätzliche Limitierungen aufweist.

Neben der schnellen und erfolgreichen Anwendung des SGA bieten Anwendungsprobleme bei der Platzierung der SGA am Phantom einen Hinweis auf potenzielle Verzögerungen bei der Anwendung am Patienten. Im Gegensatz zu LT und LMA traten bei der i‑gel deutlich häufiger Platzierungsprobleme auf. Bei einer genaueren Betrachtung der Ergebnisse erkennt man, dass am Phantom speziell das Auftreten von „Nebenluft“ (45 % der Fälle) im Rahmen der Beatmung sowie ein erhöhter Reibungswiderstand (15 % der Fälle) im Rahmen der Platzierung als Anwendungsprobleme beschrieben wurden.

Das Problem der Nebenluft ist bei der i‑gel auch im klinischen Alltag zu beobachten, bis der thermoelastische Cuff sich durch die Körperwärme des Patienten an die Strukturen im Hypopharynx angepasst hat. Die Studie von de Montblanc zeigt am anästhesierten Patienten, dass die durch uns am Phantom erhobenen Zeiten auf Patienten übertragbar sind [4], die erfassten Probleme „Nebenluft“ und „Reibungswiderstand“ jedoch in der klinischen Anwendung von geringerer Relevanz sind. Betrachtet man daher die o. g. Probleme als technische Probleme des Versuchsaufbaus, die am Patienten keine Auswirkungen haben, traten bei der i‑gel im Vergleich zu den übrigen SGA lediglich in 43 % der Anwendungen Probleme auf. Unbeantwortet muss die Frage bleiben, ob ein unerfahrener Anwender auf die Verwendung des i‑gel verzichten würde, weil er die Nebenluft irrtümlich als Zeichen einer insuffizienten Beatmung wertet. Weiterhin bleibt unklar, wie ausgeprägt der Aspirationsschutz in dieser Zeitspanne am Patienten ist.

Der mit einem thermoplastischen Gel gefüllte Cuff der i‑gel könnte jedoch gerade in Bezug auf die Anwendung durch Laien Vorteile bringen, da ein aufblasbarer Cuff in vergangenen Studien oftmals indirekt und direkt zu Komplikationen in der Anwendung geführt hat.

Wie auch in anderen Studien von Schälte et al. traten in dieser Studie Probleme mit der suffizienten Blockung des Cuffs sowie des Erreichens einer suffizienten Einführtiefe bei den verschiedenen SGA auf.

Durch den konstruktionsbedingten Wegfall des Arbeitsschritts der Luftinsufflation in einen Cuff bei der i‑gel im Vergleich zu LT und LMA entsteht zunächst eine deutliche Zeitersparnis bis zur ersten Beatmung. Weiterhin wird die Fehlerquelle bezüglich der Stärke der Cuffblockung, die sich oftmals als nicht ausreichend erwies, ausgeschaltet. Genau so können Komplikationen durch eine zu starke Blockung des Cuffs auftreten, so z. B. Schädigungen der pharyngealen und laryngealen Mukosa einhergehend mit ödematösen Veränderungen, die den Wechsel auf einen definitiven Atemweg deutlich erschweren können [2, 12, 15, 20]. Diese Komplikationen wurden bisher bei der Anwendung der i‑gel selbst bei kindlichen Patienten mit einer deutlich vulnerableren Schleimhaut nicht berichtet [9]. Lee et al. verglichen in einer randomisierten prospektiven Studie LMA Classic und die i‑gel in Bezug auf den Verschlussdruck, die Einführzeit, die Leichtigkeit der Handhabung und beobachtete Komplikationen. Sie kamen dabei zu dem Ergebnis, dass die i‑gel signifikant schneller zu platzieren ist als die LMA Classic (17 vs. 21 s, *p* = 0,002) und sich der Verschlussdruck der beiden SGA nicht signifikant voneinander unterscheidet.

In unserer Studie zeigte sich eine besonders hohe Akzeptanz der eingesetzten supraglottischen Atemwegshilfen durch die Studienteilnehmer sowie die ausgeprägte Befürwortung einer Ausbildung an SGA in Ersthelferkursen. Diese Daten bestätigen bereits gezeigte Akzeptanzwerte in anderen Studien [18]. In den Gruppen „LT“ und „i-gel“ befürworteten 100 % der Probanden das Erlernen der Beatmung mit supraglottischen Atemwegen im Ersthelferkurs, in der Gruppe „LMA“ betrug dieser Anteil 92 %.

Dies spiegelt aus Sicht der Autoren den Wunsch der Studienteilnehmer wider, im Rahmen einer Reanimationssituation grundsätzlich neben der Herzdruckmassage auch eine Beatmung durchführen zu wollen, da die Notwendigkeit einer adäquaten Oxygenierung zur Erreichung eines guten neurologischen Outcomes im Rahmen des BLS auch Laienhelfern verständlich ist. Die größten Ressentiments bestehen jedoch in hygienischer Hinsicht gegenüber der Mund-zu-Mund- bzw. Mund zu Nase-Beatmung. SGA können helfen diese Hemmschwelle zu überwinden [10, 18], da durch die Verwendung solcher Hilfsmittel der direkte Kontakt zu den Atemwegen des Patienten vermieden werden kann. Die in der Studie erhobenen Ergebnisse unterstützen somit aus Sicht der Teilnehmer die Einführung der Ausbildung an SGA in Erste-Hilfe-Kursen.

## Schlussfolgerung

Die Daten zeigen, dass die i‑gel im Vergleich zu den anderen SGA die schnellste Platzierungszeit bei gleicher Erfolgsrate aufweist. Weiterhin reduziert der thermoplastische Cuff der i‑gel im Vergleich zu LMA und LT u. a. die Probleme mit der suffizienten Blockung. Auch wenn die Übertragbarkeit der Anwendung von SGA am Phantom auf den Menschen eingeschränkt scheint oder zumindest kontrovers diskutiert wird [11, 13, 14, 16], ist eine Nutzung durch ungeübte Laien und medizinisch vorgebildete Helfer aus Sicht der Autoren sinnvoll, da hierdurch die Chance auf eine suffiziente Oxygenierung im Rahmen des BLS erhöht wird. Unbenommen davon bleibt die Notwendigkeit einer sofortigen Kontrolle des verwendeten Atemwegshilfsmittels durch das ersteintreffende Rettungsdienstpersonal im Rahmen der Übernahme der Notfallversorgung.

Eine regelmäßige Auffrischungsausbildung in lebensrettenden Sofortmaßnahmen sowie der Anwendung der i‑gel könnte zusätzliche Handlungssicherheit im Notfall bringen und Hemmschwellen weiter abbauen.

## Fazit für die Praxis

- Die Reanimation ist die häufigste Indikation für eine prähospitale Atemwegssicherung [22]
- Oft steht einem effektiven Basic-Life-Support durch Laien die Hemmschwelle zu helfen gegenüber. Diese Hemmschwelle könnte zumindest in Bezug auf die Beatmung durch den Einsatz von SGA (supraglottische Atemwegshilfe) reduziert werden.
- Die i‑gel ließ sich im Vergleich der SGA am schnellsten am Phantom platzieren; die Ausbildung von SGA wurde durch die Studienteilnehmer deutlich befürwortet.
